# Editorial: Diagnosis, treatment and prognosis of neuroendocrine neoplasms with a focus on peptide receptor radionuclide therapy (PRRT)

**DOI:** 10.3389/fendo.2026.1920964

**Published:** 2026-09-08

**Authors:** Ariadni Spyroglou, Pernille Holmager, Krystallenia I. Alexandraki

**Affiliations:** 1 Second Department of Surgery, Aretaieio Hospital, National and Kapodistrian University of Athens, Athens, Greece; 2 Department of Nephrology and Endocrinology, Copenhagen University Hospital-Rigshospitalet, Copenhagen, Denmark

**Keywords:** neuroendocrine neoplasms, peptide receptor radionuclide therapy, somatostatin analogues, α-emitter therapy, neuroendocrine tumours

## Introduction

Neuroendocrine neoplasms (NENs) arising from neuroendocrine cells can develop throughout the body from pituitary to thyroid and paraganglion or the gastroenteropancreatic (GEP) and bronchopulmonary (BP) tract (1). Focusing on the latter two origins, they can be classified by the Ki-67 proliferation index and mitotic count into distinct grades of differentiation (grade 1, grade 2 and grade 3 for GEP; typical, atypical, large-cell/small-cell lung carcinoma for BP) or by their morphology in well-differentiated neuroendocrine tumours (NET)s or poorly differentiated neuroendocrine carcinomas (NEC)s (2, 3). Molecular and biological profiles may characterize them further in the future (4). Regarding the symptoms, NENs can be functional, due to their hormonal secretion along with the respective clinical syndrome or nonfunctioning producing only symptoms by local growth or metastatic disease. Well-differentiated NENs present a rather indolent history, so that survival of patients with NENs depends not only on their biological behavior but also on the efficacy of sequential antiproliferative treatments along with comparative or combination studies.

The majority of well-differentiated NENs present positive uptake on somatostatin receptor (SSTR) imaging (SRI) and positive immunohistochemical staining for the SSTR2a and SSTR5 (5). In this context, besides surgical removal, somatostatin analogues (SSAs) are considered essential in NEN treatment, thanks to their both symptomatic and antiproliferative action (6–8). Additionally, a number of targeted molecular treatments such as mTOR inhibitors, like everolimus, and tyrosine kinase inhibitors (TKIs), like sunitinib, cabozantinib or surufatinib, can be used prior to classic cytotoxic treatment, like the combinations temozolomide/capecitabine or streptozotocin/5-fluorouracil, for the management of advanced NETs (9), while for the NEC treatment carboplatin/etoposide or cisplatin/irinotecan can be applied (10).

## Aims and objectives

In this Research Topic, we have focused on ‘Diagnosis, Treatment and Prognosis of Neuroendocrine Neoplasms with a focus on peptide receptor radionuclide therapy (PRRT) that after the NETTER-1 has been characterized as a revolutionary treatment (11–13) that can achieve disease control in well-differentiated advanced NENs but can additionally lead to symptom control in functioning NENs (14, 15). Of note, PRRT holds a benefit over its comparators such as SSAs, sunitinib (16) or everolimus, whereas more data will be available with the COMPOSE trial (NCT04919226) (17).

## Overview of contributions

Several radionuclides have been tested in radiolabeled SSA therapy, such as ^111^In-DTPA^0^-octreotide, ^90^Y-DOTA^0^-Tyr^3^–octreotide (^90^Y-DOTATOC) and ^177^Lu-DOTA^0^-Tyr^3^–octreotate (^177^Lu-DOTATATE) (12). ^177^Lu-DOTATATE efficacy was initially tested in patients with advanced midgut NETs at disease progression on SSA treatment in the NETTER-1 study, where 4 cycles of the treatment offered a significantly longer progression free survival (PFS) in comparison to the control group receiving above-label doses 60 mg Octreotide LAR monthly (12). Comparable efficacy in the PFS showed this treatment, when applied as first-line therapy in patients with well-differentiated grade 2 or 3 tumors in the NETTER-2 study (13). On top, a repetition of PRRT treatment can be considered in patients with disease progression at least one year after initial treatment (2). In this issue, the study of Mathew et al., adds more evidence for this important treatment demonstrating that patients with metastatic pancreatic NENs receiving more than four cycles of PRRT showed improved overall survival, with the best responses documented in patients with low-grade tumors and without bone metastases.

So far, several factors have been considered as possible effectors of PRRT efficacy. Tumor derived inflammatory biomarkers, the commercially available NETest and the PRRT Predictive Quotient (PPQ), the PET-based molecular tumor volume and the chromogranin A levels have been used as predictive biomarkers for PRRT responsiveness (18–20). In line with these paradigms, Jacques et al. investigated here the presence of a specific molecular pattern focusing on four mRNAs and three miRNAs. Thereby, they identified that patients with progression one year later displayed a significantly reduced miRNA/mRNA expression profile, with this signature enabling the early identification of PRRT responders.

Even though PRRT is considered a safe treatment, adverse events are emerging with use including reduced bone marrow reserves, kidney function decline mainly in patients with already impaired renal function particularly in pretreated patients or in patients with comorbidities (21–23). Taking into consideration the increased life expectancy of NEN patients and the eventual use of PRRT as a first-line therapy investigated by NETTER-3 (NCT06784752) (24), the attempt of Stemann Lau et al. to identify markers predicting kidney function decline in patients undergoing PRRT defines a timely need. The authors measured significantly lower levels of urine concentration of the degradation biomarker of type III collagen, reflecting fibrolysis (C3M) in patients with subsequent renal impairment, an important finding that needs to be explored in further studies.

In addition, the evidenced value of PRRTs in functioning NETs, is presented in the case series of Bora Cengiz et al., where two patients with pancreatic vasoactive intestinal peptide (VIP)-secreting NETs (VIPomas), that were not responsive to any other systematic or local treatments, presented a fast resolution of diarrhea and fluid and electrolyte complications only a few days after one single PRRT cycle (15). This finding supports further the value of PRRTs as a salvage treatment in the emergency of uncontrolled functional NENs (14).

Finally, given the infrequent appearance of NENs, the identification of two synchronous, non-functional NENs in the study of Ling et al. underlines the alert for the physician to consider such a diagnosis, even in this atypical clinical context of a postpartum female with intermittent abdominal pain, with negative ^68^Ga DOTANOC PET/CT lesions, rendering them inappropriate for PRRT.

## Implications and future directions

Nowadays, PRRT is offered in a standard course of 4 treatment cycles over many months (2), while, in case of disease progression at least one year after an effective treatment, treatment decisions may include repetition of PRRT (2). A meta-analysis including re-treatment studies showed a median PFS of 12.5 and a median overall survival of 26.8 months in re-treated patients (25). In a retrospective study, PRRT re-treatment with two additional cycles, in patients with metastatic, well-differentiated NENs after disease progression was well tolerated and associated with a clinically relevant PFS and overall survival (26), offering new treatment strategies in selected patients. Similarly, in a large cohort of repeated PRRT treatment, disease control rate was 89% after the first repetition with a median PFS of 19 months (27).

Furthermore, besides from the well-established treatment with the β-particle emitter ^177^Lu-DOTATATE, targeted α-emitter therapy with isotopes such as ^212^Pb-DOTAMTATE has shown a comparable safety profile with a significantly higher overall response rate in comparison to ^177^Lu-DOTATATE (54.3% in the ALPHAMEDIX 02 study vs 18% in the NETTER-1 study), rendering this molecule a promising treatment option (28). Another α-emitter, namely RYZ101 (^225^Ac-DOTATATE), has now been tested in advanced, well-differentiated NENs progressing after ^177^Lu-DOTATATE therapy and has demonstrated acceptable tolerability with promising efficacy in a phase 1b study (29).

## Call to action

Taken together, PRRT treatment offers an increasing spectrum of treatment options in both naive and pretreated patients with well-differentiated SSA-positive NENs. We hope that this Research Topic will be a valuable addendum to current knowledge regarding PRRT in NENs, and that it will inspire future research.
